# Oncological outcomes of planned and unplanned low Hartmann’s procedure and restorative low anterior resection for rectal cancer: a population-based cross-sectional study

**DOI:** 10.1007/s10151-025-03169-5

**Published:** 2025-11-23

**Authors:** E. G. M. van Geffen, F. S. Verheij, S. M. J. A. Hazen, T. C. Sluckin, E. C. J. Consten, J.-W. T. Dekker, J. Nederend, K. C. M. J. Peeters, J. H. W. de Wilt, S. van Dieren, R. Hompes, J. B. Tuynman, C. A. M. Marijnen, P. J. Tanis, M. Kusters

**Affiliations:** 1 Department of Surgery, Cancer Center Amsterdam, Amsterdam UMC Location Vrije Universiteit Amsterdam, P.O. Box 7057, 1007 MB Amsterdam, The Netherlands; 2 Treatment and Quality of Life and Imaging and Biomarkers, Cancer Centre Amsterdam, Amsterdam, The Netherlands; 3 Department of Surgical Oncology, The Netherlands Cancer Institute, Amsterdam, The Netherlands; 4 Department of Surgery, Meander Medical Centre, Amersfoort, The Netherlands; 5 Department of Surgery, Reinier de Graaf Gasthuis, Delft, The Netherlands; 6 Department of Radiology, Catharina Hospital, Eindhoven, The Netherlands; 7 Department of Surgery, Leiden University Medical Centre, Leiden, The Netherlands; 8 Department of Surgical Oncology, Radboud University Medical Centre, Nijmegen, The Netherlands; 9 Department of Radiotherapy, Leiden University Medical Centre, Leiden, The Netherlands; 10 Department of Radiotherapy, Netherlands Cancer Institute, Amsterdam, The Netherlands; 11 Department of Surgery, Amsterdam UMC Location University of Amsterdam, Amsterdam, The Netherlands; 12 Department of Surgical Oncology and Gastrointestinal Surgery, Erasmus MC, Rotterdam, The Netherlands

**Keywords:** Rectal cancer, Minimally invasive surgical procedures, Conversion to open surgery, Resection margins

## Abstract

**Background:**

In the Netherlands, approximately 15% of patients with rectal cancer undergo a low Hartmann’s procedure (low-HP). This is often preoperatively planned to avoid poor functional outcome or complications, but might be unplanned as a result of intraoperative difficulties. Low-HPs seem to be associated with worse oncological outcomes.

**Methods:**

All patients who underwent either restorative low anterior resection (rLAR), planned low-HP, or unplanned low-HP for primary rectal cancer in 2016 were included from a nationwide cohort. Main outcomes were 4-year local recurrence (LR) rate and disease-free survival (DFS).

**Results:**

Of 2043 patients, 1704 underwent rLAR (83.4%), 253 planned low-HP (12.4%), and 86 unplanned low-HP (4.2%). Among intended rLAR patients (*n* = 1790), independent risk factors for unplanned low-HP were older age, higher body mass index (BMI), higher American Society of Anesthesiologists (ASA) score, and more distal tumor location. Oncological outcomes after low-HPs were worse than after rLARs (LR 13.7% vs 5.6%, DFS 54.7% vs 71.8%, both *p* < 0.001), but similar for unplanned and planned low-HP. In multivariable analysis, unplanned and planned low-HP were not associated with LR or DFS, but R1 resection was (HR 6.6 (4.1–10.6), HR 3.0 (2.2–4.0), respectively). In R1 resections, the distal margin was more often involved after low-HP (70.0% vs 28.6%, *p* = 0.013) compared to rLAR.

**Conclusion:**

Poor outcomes in univariable analysis after low-HP appear to be associated with more challenging procedures and increased risk of involved resection margin rather than the low-HP itself. In case of expected difficulties, an extralevator abdominoperineal excision or referral to an expert center might be an alternative to improve resection margins.

**Trial registration:**

ClinicalTrials.gov, identifier NCT05539417, retrospectively registered on September 16, 2022.

**Supplementary Information:**

The online version contains supplementary material available at 10.1007/s10151-025-03169-5.

## Introduction

The type of total mesorectal excision (TME) is thought to influence oncological outcomes in patients with rectal cancer [1–3]. If a tumor is located near the anorectal junction (ARJ), a more complex TME is needed, including partial intersphincteric resection with or without anastomosis, or an abdominoperineal excision (APE) if the levator ani is involved [4]. When the location of the tumor allows for construction of an anastomosis and sphincter preservation, a low anterior resection (LAR) is performed. Most often an anastomosis is created, resulting in a restorative LAR (rLAR). Alternatively, a non-restorative procedure can be chosen, where an end-colostomy is constructed after stapling the rectum distally from the tumor, also known as low Hartmann’s procedure (low-HP) [5, 6].

The decision to perform a low-HP is generally made preoperatively. The main reasons for planned low-HP are anticipated poor functional outcome or a high risk of mortality from anastomotic leakage. However, low-HP may also result from intraoperative technical difficulties which withhold surgeons from creating an anastomosis. An unplanned low-HP might be related to a narrow pelvis, obesity, and/or a bulky distal tumour, making it more difficult to create a tension-free anastomosis [7]. As a result, patients planned for n-rLAR are expected to differ significantly from those who undergo n-rLAR unplanned, with more comorbidities, higher age, and more neoadjuvant irradiation. Social and cultural factors may also influence acceptance of a permanent ostomy [8–12]. Furthermore, a decreasing trend in the proportion of low-HPs was observed in the Netherlands, probably as a result of sub-specialization and auditing [13]. Nevertheless, a low-HP was still performed in 14% of rectal cancer resections in 2016, with interhospital variability ranging from 0 to 42% [14].

Current literature suggests that a low-HP is associated with worse oncological outcomes, with circumferential margin positivity rates reaching 32%, and a high 3-year local recurrence (LR) rate up to 15% [15, 16]. The specific factors leading to the poorer oncological outcomes of low-HP compared to rLAR remain unknown. It was hypothesized that technical difficulties during low pelvic dissection might have contributed to the intraoperative decision to perform a low-HP, resulting in an inferior TME specimen. Therefore, this study aimed to compare oncological outcomes of patients who underwent low-HP with rLAR and identify factors, including unplanned procedures, that contribute to differences in these outcomes.

## Methods

Data collection for this retrospective population-based cohort study by the Dutch Snapshot Research Group was conducted between 2020 and 2021 in the Netherlands. All patients who underwent curative resection for primary rectal cancer in 2016 were included from 67 of the 69 Dutch hospitals providing rectal cancer care. Short-term outcomes were available from registration in the Dutch ColoRectal Audit (DCRA) and additional variables and 4-year follow-up data were collected by local teams through a secured web-based tool. These collaborative teams consisted of surgical residents or physician assistants, supervised by a surgeon. A more detailed description of the study design can be found in previously published studies [17].

### Patient selection

Patients who underwent a local excision (with or without completion TME), proctocolectomy, or had unknown surgical treatment were excluded. For baseline description, both patients who underwent an APE and those who underwent (n-)rLAR as initial surgical treatment for primary rectal cancer were included, regardless of neoadjuvant treatment. After this, patients who underwent APE or had unregistered planned/unplanned low-HP were excluded (Online Appendix 1).

### Rectal cancer treatment according to Dutch guidelines

In 2016, the Dutch national guideline recommended neoadjuvant chemoradiation for patients with advanced rectal cancer (cT4, MRF+, cN2, or pathological extramesorectal nodes). For intermediate tumors (cT3cdN0 or cT1–3N1(MRF−)), shared decision-making was advised regarding the choice between TME alone or short-course radiotherapy. In case of low-risk rectal cancer (cT1–cT3abN0) neoadjuvant treatment was not recommended. At that time, total neoadjuvant therapy, whether with induction or consolidation chemotherapy, was not part of routine practice. Following neoadjuvant treatment, patients were counselled regarding the intended surgical approach. As part of this counselling, they were also informed about the risk of intraoperative deviation from the initial surgical plan—specifically, the possibility that a restorative procedure might not be feasible and that formation of an ostomy might be required.

### Outcome measures and definitions

The primary outcome was 4-year LR rate. Secondary outcomes included metachronous distant metastases (DM), disease-free survival (DFS), overall survival (OS), and conversion rate from rLAR to low-HP. Moreover, R1 resection rate and type of positive margin (circumferential or distal) were evaluated.

An rLAR was defined as a rectal resection with a colorectal or coloanal anastomosis, with or without diverting stoma. Low-HP was defined as rectal resection with stapling of the rectal stump and formation of an end colostomy, leaving the rectal stump in situ. The decisions on the type of surgical resections were made during multidisciplinary meetings. Data regarding surgeon experience were not available. Conversion from r-LAR to low-HP was collected from the operation report. Rationale for conversion to low-HP was not collected. Tumor location was measured from its distal border to the anorectal junction (ARJ). Metachronous DMs were those developed after the 3-month postoperative period; those that occurred within 3 months were categorized as synchronous. Pelvic sepsis was defined as presacral abscess or anastomotic leakage according to any of the following: contrast extravasation on imaging studies, a presacral abscess collection requiring surgical, radiological or endoscopic intervention, or a presacral collection that either led to delay in ostomy closure or to resection of the colorectal anastomosis. A positive resection margin (R1) was defined as a pathological circumferential or distal resection margin (CRM/DRM) of 1 mm or less.

### Statistical analysis

Categorical data are presented as numbers and percentages and compared with a *χ*^2^ test. Continuous data are presented as mean with standard deviation or median with interquartile range, and compared with an independent *t* test, Mann–Whitney *U* test or one-way analysis of variance (ANOVA). Missing data were reported, and analyses were performed using complete cases. Logistic regression assessed variables associated with the risk of low-HP in patients undergoing intentional rLAR. Univariable variables with *p* < 0.10 were selected for multivariable regression using backwards selection. Kaplan–Meier estimates were used for 4-year DFS, OS, LR, and DM rates and compared with a log-rank test. Multivariable Cox proportional hazard evaluated the association between surgery type and oncological outcomes. A mediation and sensitivity analysis was applied, removing surgery type (minimally invasive, minimally invasive converted, and open) and adding the APE cohort, to demonstrate sensitivity to cohort characteristics and understand the causal mechanism. Analyses were conducted using IBM SPSS statistics version 28 (Chicago, IL), with a two-sided *p* value < 0.05 considered statistically significant.

### Ethics

The Medical Ethics Committee of the Amsterdam UMC approved this study and determined it to be exempt from the Dutch Medical Research Involving Human Subjects Act. Local institutional review boards approved the study execution and determined patient consent procedures.

## Results

In total 2772 primary TME procedures were performed in 2016 (Supplementary Fig. 1), with a median follow-up period of 49 months (IQR 34–55). An APE was performed in 699 patients (24.6%), a rLAR in 1704 patients (61.5%), and a low-HP in 369 patients (13.9%).

An APE was performed in patients with more distal and more locally advanced tumors, who also received neoadjuvant chemoradiation more often, compared to (n-)rLAR cases, and there were more R1 resections in the APE group (Table 1). Patients who underwent low-HP were older than rLAR patients (73 vs 65 years old, *p* < 0.001), had a prior bowel resection more often (3.5% vs 0.8%, *p* < 0.001), a higher ASA score (ASA III/IV 32.5% vs 13.8%, *p* < 0.001), and presented with more distally located tumors, higher clinical T stage (cT stage), and more often with synchronous metastases (Table 1). The surgical procedure was open in 22.5% of low-HP vs 8.9% of rLAR (*p* < 0.001), and postoperative blood transfusions were more common after low-HP (15.3% vs 4.7% in rLAR, *p* < 0.001). Moreover, in low-HP there were more R1 resections (10.0% vs 2.9% in rLAR, *p* < 0.001).

**Table 1:** Patient, tumor, operative, and histopathological details according to type of procedure

	Whole cohort (*N* = 2772)	APE (*N* = 699; 24.6%)	rLAR (*N* = 1704; 61.5%)	Low-HP (*N* = 369; 13.9%)	*p* value APE vs rLAR vs low-HP	*p* value rLAR vs low-HP
Sex, male	1789 (64.5)	469 (67.1)	1099 (64.5)	221 (59.9)	0.065	0.095
Age at resection, mean (SD)	66.9 (10.2)	67.2 (10.5)	65.4 (9.6)	73.3 (9.7)	< 0.001*	< 0.001*
BMI, mean (SD)	26.5 (4.4)	26.7 (4.7)	26.4 (4.2)	26.6 (4.5)	0.156	0.590
Previous bowel segment resection	49 (1.8)	22 (3.1)	14 (0.8)	13 (3.5)	< 0.001*	< 0.001*
ASA III/IV	492 (17.7)	139 (20.0)	233 (13.8)	120 (32.5)	< 0.001*	< 0.001*
Distance from the ARJ in cm, mean (SD)	5.3 (3.6)	1.7 (2.1)	6.8 (3.1)	5.2 (2.8)	< 0.001*	< 0.001*
cT stage					< 0.001*	< 0.001*
cT1/T2	764 (27.6)	159 (23.2)	524 (32.1)	81 (22.7)		
cT3	1617 (58.3)	389 (56.8)	997 (61.1)	231 (64.7)		
cT4	269 (9.7)	131 (19.1)	96 (5.9)	42 (11.8)		
cTx	25 (0.9)	6 (0.9)	16 (1.0)	3 (0.8)		
MRF^a^	993 (61.4)	161 (43.2)	691 (70.7)	141 (62.9)	< 0.001*	0.030*
cN stage					< 0.001*	< 0.001*
cN0	1110 (41.5)	255 (37.2)	731 (44.8)	124 (34.7)		
cN1	862 (32.2)	203 (29.6)	537 (32.9)	122 (34.2)		
cN2	691 (25.8)	224 (32.7)	358 (21.9)	109 (29.5)		
cNx	12 (0.4)	3 (0.4)	7 (0.4)	2 (0.6)		
Synchronous metastases	204 (7.4)	60 (8.6)	105 (6.2)	39 (10.6)	0.005*	0.003*
Neoadjuvant radiotherapy					< 0.001*	< 0.001*
None	1071 (38.6)	176 (25.2)	761 (44.7)	134 (36.3)		
5 × 5 short interval	471 (17.0)	79 (11.3)	334 (19.6)	58 (15.7)		
5 × 5 long interval	298 (10.8)	94 (13.4)	145 (8.5)	59 (16.0)		
Chemoradiation	932 (33.6)	350 (50.1)	464 (27.2)	118 (32.0)		
Multivisceral resection	215 (7.8)	101 (14.4)	78 (4.6)	36 (9.8)	< 0.001*	< 0.001*
Initial approach					< 0.001*	< 0.001*
Open	381 (13.7)	146 (20.9)	152 (8.9)	83 (22.5)		
Laparoscopic	1944 (70.1)	464 (66.4)	1220 (71.6)	260 (70.5)		
TaTME	169 (6.1)	24 (3.4)	136 (8.0)	9 (2.4)		
Robot	269 (9.7)	65 (9.3)	189 (11.1)	15 (4.1)		
Other	9 (0.4)	0 (0.0)	7 (0.5)	2 (0.6)		
Conversion to open surgery^b^	109 (4.6)	22 (4.0)	69 (4.5)	18 (6.4)	0.279	0.170
Duration of surgery (min), mean (SD)	210.3 (92.0)	246.3 (106.7)	197.1 (78.9)	198.6 (96.3)	< 0.001*	0.795
Blood transfusion	221 (8.4)	92 (13.6)	76 (4.7)	53 (15.3)	< 0.001*	< 0.001*
Complications within 30 days of surgery	1027 (37.4)	288 (41.2)	607 (36.0)	132 (36.1)	0.126	0.897
Surgical complications^c^	666 (64.8)	172 (59.7)	414 (68.2)	80 (60.6)	0.025*	0.093
Reinterventions^d^	351 (52.6	73 (42.4)	241 (58.2)	37 (46.3)	0.001*	0.048*
Pelvic sepsis	404 (14.6)	72 (10.3)	291 (17.1)	66 (17.9)	< 0.001*	0.709
Readmitted	694 (25.0)	155 (22.2)	450 (26.4)	89 (24.1)	0.085	0.395
Number of readmissions, mean (SD)	1.65 (1.41)	1.53 (1.19)	1.69 (1.40)	1.77 (1.83)	0.319	0.715
(y)pT stage					< 0.001*	< 0.001*
(y)pT0	206 (7.4)	62 (8.9)	123 (7.2)	21 (5.7)		
(y)pT1	323 (11.7)	69 (9.9)	233 (13.1)	31 (8.4)		
(y)pT2	860 (31.0)	223 (31.9)	540 (31.7	97 (26.3)		
(y)pT3	1264 (45.6)	308 (43.1)	764 (44.8)	192 (52.0)		
(y)pT4	116 (4.2)	37 (5.3)	51 (3.0)	28 (7.6)		
(y)pTx	3 (0.1)	0 (0.0)	3 (0.2)	0 (0.0)		
(y)pN stage					0.214	0.067
(y)pN0	1825 (65.8)	465 (66.5)	1137 (66.7)	223 (60.4)		
(y)pN1	647 (23.3)	157 (22.5)	390 (22.9)	100 (27.1)		
(y)pN2	298 (10.8)	76 (10.9)	176 (10.3)	46 (12.5)		
(y)pNx	2 (0.1)	1 (0.1)	1 (0.1)	0 (0.0)		
Involved resection margin	166 (6.0)	80 (11.5)	49 (2.9)	37 (10.0)	< 0.001*	< 0.001*

Data are presented as* n* (%), unless otherwise stated

*APE* abdominoperineal excision, *rLAR* restorative lower anterior resection, *low-HP* non-restorative lower anterior resection, *SD* standard deviation, *BMI* body mass index, *ASA* American Society of Anesthesiologists, *ARJ* anorectal junction, *cT stage* clinical T stage, *MRF* mesorectal fascia, *cN stage* clinical N stage, *TaTME* transanal total mesorectal excision, *min* minutes, *(y)pT stage* (post-therapy) pathological T stage, *(y)pN stage* (post-therapy) pathological N stage

^a^As percentage of the cT3 tumors

^b^As percentage of the laparoscopic, TaTME, and robot approach

^c^As percentage of total complications < 30 days

^d^As percentage of surgical complications < 30 days

*Statistically significant (*p* < 0.05)

### Planned vs unplanned low-HP

After excluding APE patients and 46 patients who underwent low-HP with unknown planning status, a total of 2043 patients could be included for final analyses. rLAR was performed in 1704 (83.4%), planned low-HP in 253 (12.4%), and unplanned low-HP in 86 patients (4.2%). The proportion of unplanned low-HP among patients who underwent low-HP was 86/339 (25.4%).

Patients who underwent a planned low-HP were older compared to patients in the unplanned low-HP group (74 vs 70 years old, *p* < 0.001, Table 2). Preoperative tumor characteristics, including distance to the ARJ and cT/N stage were not statistically different between both groups. While intentional minimally invasive approach was similar (planned 79.5% vs unplanned 71.4%, *p* = 0.185), the percentage of conversion to open surgery was significantly higher in the unplanned group compared to the planned group (17.5% vs 3.0%, *p* < 0.001). Moreover, the mean duration of surgery was longer for unplanned low-HPs compared to planned procedures (246 vs 181 min, *p* < 0.001).

**Table 2:** Patient, tumor, operative, and histopathological details according to type of low-HP

	rLAR (*N* = 1704)	Unplanned low-HP (*N* = 86)	Planned low-HP (*N* = 253)	*p* value rLAR vs unplanned low-HP	*p* value Planned vs unplanned low-HP
Sex, male	1099 (64.5)	58 (67.4)	147 (58.1)	0.644	0.126
Age at resection, mean (SD)	65.4 (9.6)	69.7 (9.9)	74.4 (9.2)	< 0.001*	< 0.001*
BMI, mean (SD)	26.4 (4.2)	27.2 (5.5)	26.4 (4.2)	0.225	0.230
Previous bowel segment resection	14 (0.8)	5 (5.8)	6 (2.4)	< 0.001*	0.055
ASA III/IV	233 (13.8)	28 (32.6)	87 (34.5)	< 0.001*	0.842
Distance from the ARJ in cm, mean (SD)	6.8 (3.1)	4.9 (2.6)	5.3 (2.8)	< 0.001*	0.289
cT stage				0.262	0.770
cT1/T2	524 (32.1)	20 (24.4)	58 (23.7)		
cT3	997 (61.1)	54 (65.9)	157 (64.1)		
cT4	96 (5.9)	7 (8.5)	28 (11.4)		
cTx	16 (1.0)	1 (1.2)	2 (0.8)		
MRF^a^	691 (70.7)	37 (69.8)	95 (60.5)	0.896	0.388
cN stage				0.155	0.915
cN0	731 (44.8)	31 (36.6)	87 (35.5)		
cN1	537 (32.9)	27 (32.9)	86 (35.1)		
cN2	358 (21.9)	25 (30.5)	70 (28.6)		
cNx	7 (0.4)	0 (0.0)	2 (0.8)		
Synchronous metastases	105 (6.2)	13 (15.1)	23 (9.1)	0.003*	0.117
Neoadjuvant radiotherapy				0.212	0.340
None	761 (44.7)	34 (39.5)	97 (38.3)		
5 × 5 short interval	334 (19.6)	12 (14.0)	40 (15.8)		
5 × 5 long interval	145 (8.5)	9 (10.5)	44 (17.4)		
Chemoradiation	464 (27.2)	31 (36.0)	72 (28.5)		
Multivisceral resection	78 (4.6)	9 (10.5)	20 (7.9)	0.013*	0.463
Initial approach				< 0.001*	0.185
Open	152 (8.9)	22 (25.6)	51 (20.2)		
Laparoscopic	1220 (71.6)	55 (64.0)	188 (74.3)		
TaTME	136 (8.0)	4 (4.7)	4 (1.6)		
Robot	189 (11.1)	4 (4.7)	9 (3.6)		
Other	7 (0.5)	1 (1.2)	1 (0.4)		
Conversion to open surgery^b^	69 (4.5)	11 (17.5)	6 (3.0)	< 0.001*	< 0.001*
Duration of surgery (min), mean (SD)	197.1 (78.9)	246.0 (100.1)	181.4 (90.4)	< 0.001*	< 0.001*
Blood transfusion	76 (4.7)	17 (21.3)	32 (13.6)	< 0.001*	0.101
Complications within 30 days of surgery	607 (36.0)	36 (42.9)	85 (33.7)	0.430	0.131
Surgical complications^c^	414 (68.2)	24 (66.7)	49 (57.6)	0.847	0.354
Reinterventions^d^	241 (58.2)	15 (62.5)	20 (40.8)	0.679	0.081
Pelvic sepsis	291 (17.1)	18 (20.9)	40 (15.8)	0.379	0.276
Readmitted	450 (26.4)	23 (26.7)	57 (22.5)	0.945	0.427
Number of readmissions, mean (SD)	1.7 (1.40)	1.9 (1.4)	1.5 (1.3)	0.581	0.315
(y)pT stage				0.007*	0.824
(y)pT0	123 (7.2)	6 (7.0)	14 (5.5)		
(y)pT1	233 (13.1)	6 (7.0)	25 (9.9)		
(y)pT2	540 (31.7)	22 (25.6)	70 (27.7)		
(y)pT3	764 (44.8)	44 (51.2)	127 (50.2)		
(y)pT4	51 (3.0)	8 (9.2)	17 (6.7)		
(y)pTx	3 (0.2)				
(y)pN stage				0.170	0.695
(y)pN0	1137 (66.7)	49 (57.0)	156 (61.7)		
(y)pN1	390 (22.9)	26 (30.2)	65 (25.7)		
(y)pN2	176 (10.3)	11 (12.8)	32 (12.6)		
(y)pNx	1 (0.1)	0 (0.0)	0 (0.0)		
Involved resection margin	49 (2.9)	10 (11.6)	22 (8.7)	< 0.001*	0.422
CRM positive^e^	39 (79.6)	5 (50.0)	13 (56.5)	0.050	0.730
DRM positive^e^	14 (28.6)	7 (70.0)	12 (52.2)	0.013*	0.341

Data are presented as* n* (%), unless otherwise stated

*rLAR* restorative lower anterior resection, *low-HP* non-restorative lower anterior resection, *SD* standard deviation, *BMI* body mass index, *ASA* American Society of Anesthesiologists, *ARJ* anorectal junction, *cT stage* clinical T stage, *MRF* mesorectal fascia, *cN stage* clinical N stage, *TaTME* transanal total mesorectal excision, *min* minutes, *(y)pT stage* (post-therapy) pathological T stage, *(y)nT stage* (post-therapy) pathological N stage, *CRM* circumferential resection margin, *DRM* distal resection margin

^a^As percentage of the cT3 tumors

^b^As percentage of the laparoscopic, TaTME, and robot approach

^c^As percentage of total complications < 30 days

^d^As percentage of surgical complications < 30 days

^e^As percentage of involved resection margin

*Statistically significant (*p* < 0.05)

### Risk of unplanned low-HP

In 1790 patients, the initial surgical plan was to perform rLAR. This changed intraoperatively to a low-HP in 86 patients (4.8%). Patients who underwent unplanned low-HP were older and had a higher ASA score compared to patients who underwent rLAR (Table 2). Tumors in the unplanned low-HP group were located closer to the ARJ compared to rLAR cases (4.9 cm vs 6.8 cm,* p* < 0.001), and multivisceral resections were performed more often (10.5% vs 4.6%,* p* = 0.013). Low-HP was more often performed using a primary open approach (25.6% vs 8.9% rLAR, *p* < 0.001), and in case of primarily minimally invasive, more often converted to an open procedure (17.5% vs 4.5% rLAR). Moreover, procedures low-HP procedures took longer (246 vs 197 min, *p* < 0.001) and more often required blood transfusions postoperatively (21.3% vs 4.7%), compared to rLAR. Locally advanced disease ((y)pT4 9.2% vs 3.0%, *p* = 0.007) and involved resection margin (R1) (11.6% vs 2.9%, *p* < 0.001) were more common in the unplanned low-HP group. In case of an R1 resection, the DRM was more often involved in case of an unplanned low-HP as compared to rLAR (70.0% vs 28.6%, *p* = 0.013).

In binary logistic regression analysis including all patients who intentionally underwent rLAR (Table 3), conversion to low-HP was associated with an age above 65 years (OR 1.9 (95% CI 1.1–3.2), *p* = 0.013), a BMI of 30 or higher (OR 1.9 (95% CI 1.1–3.4), *p* = 0.013), a higher ASA classification (ASA III/IV; OR 3.1 (95% CI 1.9–5.3), *p* < 0.001), and a more distal location (ARJ < 3.0 cm; OR 8.9 (95% CI 4.2–19.0), *p* < 0.001).

**Table 3:** Uni- and multivariate binary logistic regression for conversion to low-HP

Variable	Categories	Univariable		Multivariable	
		OR (95% CI)	*P*	OR (95% CI)	*P*
Age	≤ 65	1.000	< 0.001*	1.000	0.013*
	> 65	2.176 (1.360–3.481)		1.905 (1.143–3.175)	
Sex	Male	1.000	0.577		
	Female	0.887 (0.553–1.392)			
BMI (kg/m^2^)	< 25.0	1.000	0.049*	1.000	0.013*
	25.0–30.0	0.798 (0.475–1.342)		0.788 (0.448–1.385)	
	≥ 30.0	1.619 (0.934–2.806)		1.915 (1.062–3.452)	
ASA	I/II	1.000	< 0.001*	1.000	< 0.001*
	III/IV/V	3.120 (1.942–5.014)		3.120 (1.836–5.304)	
Distance to the ARJ (cm)	> 7 cm	1.000	< 0.001*	1.000	< 0.001*
	3.1–7 cm	3.643 (1.916–6.925)		3.892 (2.017–7.509)	
	≤ 3 cm	6.890 (3.302–14.380)		8.892 (4.167–18.972)	
cT stage	T1–2	1.000	0.268		
	T3	1.419 (0.840–2.396)			
	T4	1.910 (0.786–4.642)			
cN stage	N0	1.000	0.160		
	N1	1.225 (0.720–2.085)			
	N2	1.702 (0.986–2.936)			
Neoadjuvant radiotherapy	None	1.000	0.219		
	5 × 5 short interval	0.804 (0.411–1.572)			
	5 × 5 long interval	1.389 (0.652–2.958)			
	Chemoradiation	1.495 (0.907–2.466)			

*n-rLAR* non-restorative lower anterior resection, *BMI* body mass index, *ASA* American Society of Anesthesiologists, *ARJ* anorectal junction, *cT stage* clinical T stage, *cN stage* clinical N stage

*Statistically significant (*p* < 0.05)

### Oncological outcomes according to type of procedure

**Fig. 1:**
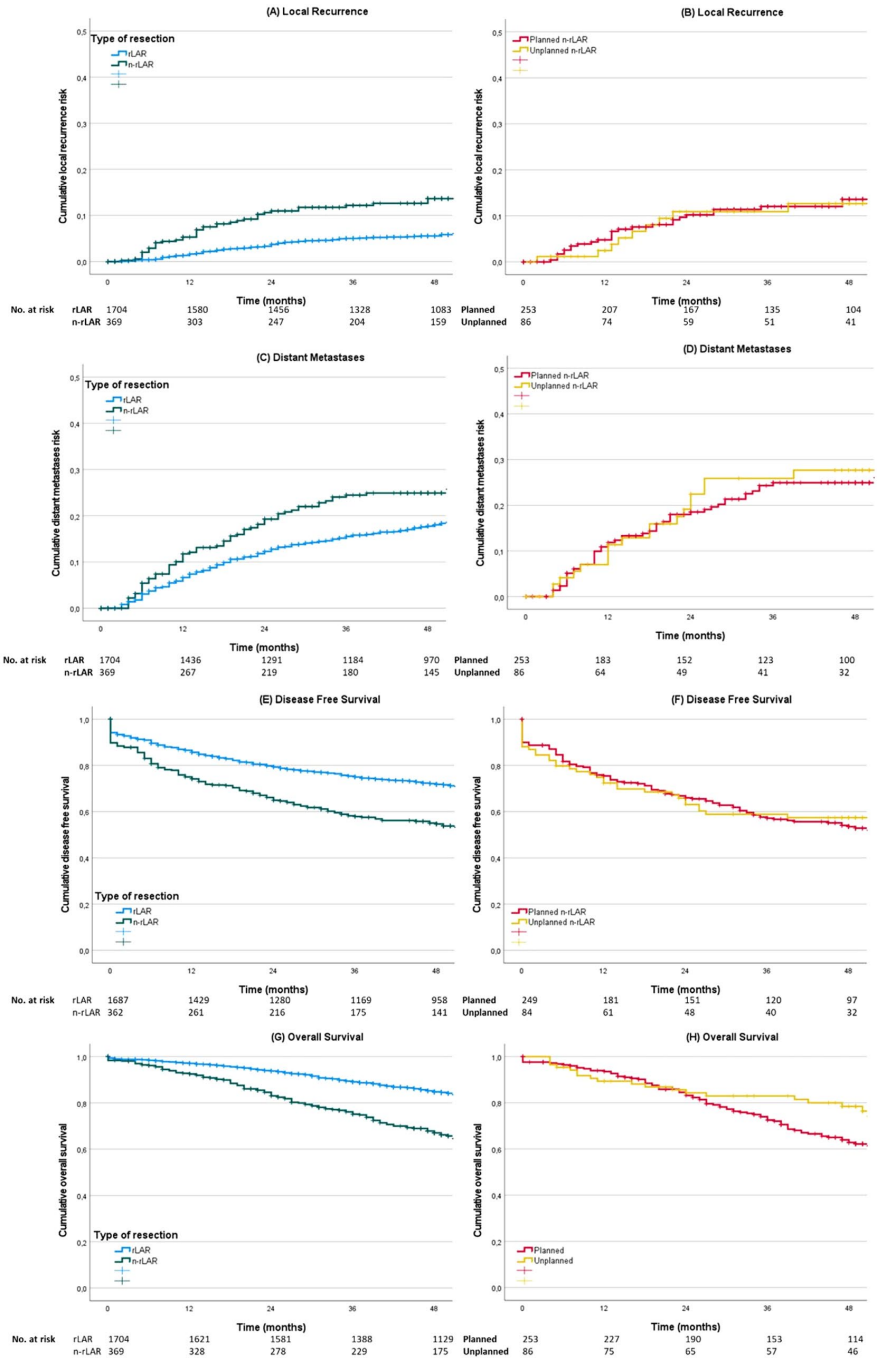
Oncological outcomes according to type of resection. **a** 4-year local recurrence rate for rLAR (5.6%) vs low-HP (13.7%, *p* < 0.001). **b** 4-year local recurrence rate for planned low-HP (12.7%) vs unplanned low-HP (13.6%, *p* = 0.852). **c** 4-year distant metastasis rate for rLAR (17.8%) vs low-HP (24.9%, *p* < 0.001). **d** 4-year distant metastasis rate for planned low-HP (24.9%) vs unplanned low-HP (27.7%, *p* = 0.718). **e** 4-year disease-free survival for rLAR (71.8%) vs low-HP (54.7%, *p* < 0.001). **f** 4-year disease-free survival for planned low-HP (53.5%) vs unplanned low-HP (57.4%, *p* = 0.845). **g** 4-year overall survival for rLAR (84.8%) vs low-HP (67.1%, *p* < 0.001). **h** 4-year overall survival for planned low-HP (78.4%) vs unplanned low-HP (62.8%, *p* = 0.064).* rLAR* restorative lower anterior resection,* n-rLAR* non-restorative lower anterior resection

At 4 years after primary rectal cancer resection, oncological outcomes were significantly worse for low-HP compared to rLAR (LR 13.7% vs 5.6%, *p* < 0.001, DM 24.9% vs 17.8%, *p* < 0.001, DFS 54.7% vs 71.8%,* p* < 0.001, and OS 67.1% vs 84.8%, *p* < 0.001, Fig. 1a–d). Within the low-HP group, cumulative incidence of LR (12.7% vs 13.6%, *p* = 0.852), DM (24.9% vs 27.7%, *p* = 0.718), DFS (53.5% vs 57.4%, *p* = 0.845), and OS (78.4% vs 62.8%, *p* = 0.064) at 4 years were not statistically different for planned and unplanned low-HP (Fig. 1e–h). After correction for covariates in a multivariable model, the type of procedure (rLAR, unplanned or planned low-HP) was not significantly associated with 4-year LR rate, DM rate, DFS nor OS (Table 4). In a second multivariable model (Supplementary Table 1A-B) in which patients who underwent APE were included and the approach (minimally invasive, minimally invasive converted, or open) was not incorporated in the model, low-HP was significantly associated with increased LR rate and DM rate and lower OS and DFS.

**Table 4:** Uni- and multivariable Cox regression analyses for (top) local recurrence and distant metastases, (bottom) disease-free survival and overall survival

	Variable	Number	Local recurrence				Distant metastases			
			Univariable		Multivariable		Univariable		Multivariable	
			HR (95% CI)	*p*	HR	*p*	HR (95% CI)	*p*	HR (95% CI)	*p*
Age	≤ 65	905	1.000	0.904			1.000	0.784		
	> 65	1168	1.021 (0.724–1.441)				1.030 (0.834–1.271)			
Sex	Male	1320	1.000	0.200			1.000	0.454		
	Female	753	0.788 (0.545–1.140)				0.919 (0.738–1.145)			
ASA	I/II	1700	1.000	0.964			1.000	0.010*	1.000	0.034*
	III/IV/V	353	1.011 (0.628–1.628)				1.430 (1.100–1.859)		1.349 (1.023–1.779)	
Distance to the ARJ	≤ 3 cm	250	1.000	< 0.001*	1.000	< 0.001*	1.000	0.118		
	3.1–7 cm	910	0.436 (0.284–0.667)		0.520 (0.338–0.800)		0.726 (0.529–0.994)			
	> 7 cm	757	0.317 (0.197–0.509)		0.405 (0.249–0.658)		0.840 (0.612–1.154)			
cT	T1–2	605	1.000	0.046*		NS	1.000	< 0.001*	1.000	0.045*
	T3	1228	1.215 (0.804–1.835)				1.871 (1.428–2.451)		1.482 (1.086–2.022)	
	T4	138	2.281 (1.281–4.212)				2.858 (1.894–4.314)		1.380 (0.826–2.306)	
MRF	Not involved	1392	1.000	0.002*		NS	1.000	< 0.001*	1.000	0.007*
	Involved	510	1.816 (1.257–2.624)				1.866 (1.487–2.341)		1.478 (1.110–1.967)	
cN	N0	855	1.000	< 0.001*		NS	1.000	< 0.001*		NS
	N1	659	0.651 (0.407–1.043)				1.533 (1.193–1.969)			
	N2	467	1.655 (1.113–2.460)				1.597 (1.215–2.099)			
Synchronous metastases	No	1929	1.000	0.010*		NS	NA			
	Yes	144	2.240 (1.285–3.904)							
Neoadjuvant radiotherapy	None	895	1.000	< 0.001*	1.000	< 0.001*	1.000	< 0.001*	1.000	< 0.001*
	5 × 5 short interval	392	0.204 (0.082–0.510)		0.163 (0.064–0.414)		1.414 (1.050–1.904)		1.145 (0.825–1.590)	
	5 × 5 long interval	204	2.132 (1.298–3.501)		1.529 (0.912–2.565)		3.362 (2.432–4.649)		2.107 (1.443–3.078)	
	Chemoradiation	582	1.428 (0.972–2.098)		1.039 (0.683–1.581)		1.649 (1.271–2.139)		1.000 (0.714–1.402)	
Type of resection	rLAR	1704	1.000	< 0.001*		NS	1.000	0.002*		NS
	Planned n-rLAR	253	2.475 (1.609–3.807)				1.548 (1.151–2.082)			
	Unplanned n-rLAR	86	2.286 (1.152–4.536)				1.700 (1.068–2.706)			
Approach	MI	1745	1.000	< 0.001*	1.000	< 0.001*	1.000	< 0.001*	1.000	0.038*
	Open	234	2.971 (1.953–4.519)		2.288 (1.453–3.603)		1.955 (1.453–2.630)		1.456 (1.026–2.067)	
	MI converted	94	2.889 (1.615–5.171)		3.452 (1.894–6.140)		1.564 (1.022–2.393)		1.452 (0.938–2.248)	
Multivisceral resection	No	1959	1.000	< 0.001*		NS	1.000	< 0.001*		NS
	Yes	114	2.913 (1.726–4.916)				2.118 (1.439–3.116)			
Duration of surgery	≤ 200 min	946	1.000	0.092		NS	1.000	0.201		
	> 200	642	1.400 (0.948–2.068)				1.168 (0.921–1.481)			
Pelvic sepsis	No	1716	1.000	0.012*	1.000	0.028*	1.000	0.224		
	Yes	357	1.656 (1.116–2.457)		1.579 (1.049–2.376)		1.179 (0.904–1.539)			
Blood transfusion	No	1830	1.000	0.357			1.000	0.316		
	Yes	129	1.375 (0.698–2.709)				1.263 (0.812–1.965)			
Margin status	R0	1986	1.000	< 0.001*	6.570 (4.088–10.559)	< 0.001*	1.000	< 0.001*	1.000	< 0.001*
	R1/R2	86	7.317 (4.660–11.489)				3.607 (2.480–5.248)		3.033 (2.033–4.526)	

	Variable	Number	Disease-free survival				Overall survival			
			Univariable		Multivariable		Univariable		Multivariable	
			HR	*p*	HR	*p*	HR (95% CI)	*p*	HR (95% CI)	*p*
Age	≤ 65	894	1.000	0.006*		NS	1.000	< 0.001*	1.000	< 0.001*
	> 65	1155	1.248 (1.065–1.462)				1.790 (1.447–2.215)		1.723 (1.360–2.182)	
Sex	Male	1299	1.000	0.123			1.000	0.194		
	Female	750	0.880 (0.747–1.035)				0.871 (0.705–1.075)			
ASA	I/II	1676	1.000	< 0.001*	1.000	< 0.001*	1.000	< 0.001*	1.000	< 0.001*
	III/IV/V	353	1.682 (1.492–2.018)		1.452 (1.192–1.768)		2.423 (1.948–3.015)		1.949 (1.523–2.493)	
Distance to the ARJ	≤ 3 cm	240	1.000	0.081		NS	1.000	0.060	1.000	0.026*
	3.1–7 cm	901	0.762 (0.600–0.966)				0.693 (0.513–0.935)		0.701 (0.511–0.961)	
	> 7 cm	752	0.815 (0.640–1.037)				0.797 (0.589–1.079)		0.919 (0.666–1.268)	
cT	T1–2	597	1.000	< 0.001*	1.000	< 0.001*	1.000	< 0.001*	1.000	< 0.001*
	T3	1216	1.885 (1.539–2.308)		1.557 (1.226–1.975)		2.002 (1.529–2.623)		1.704 (1.238–2.346)	
	T4	134	2.733 (2.005–3.727)		1.290 (0.874–1.903)		2.604 (1.732–3.913)		1.011 (0.596–1.716)	
MRF	Not involved	1378	1.000	< 0.001*	1.000	0.019*	1.000	< 0.001*		NS
	Involved	500	1.810 (1.529–2.143)		1.290 (1.042–1.598)		1.759 (1.417–2.184)			
cN	N0	841	1.000	< 0.001*		NS	1.000	< 0.001*		NS
	N1	658	1.570 (1.299–1.897)				1.485 (1.164–1.895)			
	N2	458	1.756 (1.433–2.152)				1.522 (1.169–1.981)			
Synchronous metastases	No	1905	NA				1.000	< 0.001*	1.000	< 0.001*
	Yes	144					5.444 (4.266–6.949)		3.465 (2.593–4.631)	
Neoadjuvant radiotherapy	None	883	1.000	< 0.001*	1.000	< 0.001*	1.000	< 0.001*	1.000	0.029*
	5 × 5 short interval	391	1.109 (0.879–1.400)							
	5 × 5 long interval	201	3.512 (2.807–4.394)		0.965 (0.747–1.247)		0.931 (0.681–1.273)		0.927 (0.655–1.314)	
							3.220 (2.435–4.256)			
	Chemoradiation	574	1.540 (1.271–1.865)		2.217 (1.687–2.912)		1.430 (1.118–1.828)		1.562 (1.097–2.226)	
					1.021 (0.794–1.316)				1.077 (0.792–1.464)	
Type of resection	rLAR	1687	1.000	< 0.001*		NS	1.000	< 0.001*		NS
	Planned low-HP	249	1.867 (1.521–2.292)				2.740 (2.154–3.487)			
	Unplanned low-HP	84	1.795 (1.279–2.519)				1.753 (1.124–2.732)			
Approach	MI	1727	1.000	< 0.001*	1.000	< 0.001*	1.000	< 0.001*	1.000	0.005*
	Open	231	2.380 (1.951–2.903)		1.813 (1.435–2.291)		2.850 (2.247–3.615)		1.671 (1.225–2.281)	
	MI converted	91	1.158 (0.801–1.674)		1.063 (0.724–1.561)		1.188 (0.738–1.914)		1.164 (0.707–1.915)	
Multivisceral resection	No	1938	1.000	< 0.001*		NS	1.000	< 0.001*	1.000	0.050*
	Yes	111	2.357 (1.810–3.070)				2.675 (1.954–3.661)		1.548 (1.000–2.695)	
Duration of surgery	≤ 200 min	937	1.000	0.012*		NS	1.000	0.180		
	> 200	636	1.255 (1.052–1.498)				1.171 (0.930–1.473)			
Pelvic sepsis	No	1698	1.000	0.012*	1.000	0.033*	1.000	0.003*	1.000	0.023*
	Yes	351	1.399 (1.160–1.668)		1.242 (1.018–1.516)		1.428 (1.126–1.810)		1.353 (1.043–1.755)	
Blood transfusion	No	1806	1.000	< 0.001*		NS	1.000	< 0.001*	1.000	< 0.001*
	Yes	129	1.871 (1.433–2.443)				2.996 (2.228–4.028)		1.924 (1.374–2.695)	
Margin status	R0	1956	1.000	< 0.001*	1.000	< 0.001*	1.000	< 0.001*	1.000	< 0.001*
	R1/R2	83	3.752 (2.879–4.890)		2.970 (2.229–3.958)		4.438 (3.259–6.042)		2.674 (1.889–3.784)	

*ASA* American Society of Anesthesiologists, *ARJ* anorectal junction, *cT stage* clinical T stage, *MRF* mesorectal fascia, *cN stage* clinical N stage, *rLAR* restorative lower anterior resection, *low-HP* non-restorative lower anterior resection, *MI* minimally invasive, *HR* hazard ratio

*Statistically significant (*p* < 0.05)

In this first multivariable model (Table 4), R1 resection had the strongest association with LR (HR 6.5, 95% CI 4.1–10.6, *p* < 0.001), DM (HR 3.0, 95% CI 2.0–4.5, *p* < 0.001), DFS (HR 3.0, 95% CI 2.2–4.0, *p* < 0.001), and OS (2.7, 95% CI 1.9–3.8, *p* < 0.001). In addition to R1 resection, neoadjuvant therapy and approach (minimally invasive converted to open or open approach) were strongly associated with all oncological endpoints (Table 4).

The patient characteristics of the nine patients who developed local recurrence after unplanned low-HP are shown in Supplementary Table 2; five of nine had a tumor located > 3 cm from the ARJ, three of nine did not receive neoadjuvant (C)RT; and none had a pT4 tumor.

## Discussion

In this cross-sectional national cohort involving 67 Dutch hospitals, low-HP comprised 14% of all rectal cancer resections. Among patients who underwent low-HP, the procedure was unplanned in 25%, which was 5% of the patients who were intended to undergo rLAR. Intraoperative conversion from rLAR to a low-HP was associated with higher age, higher BMI, ASA classification > 2, and a more distal tumor location. As compared to rLAR, unplanned low-HP more often resulted in resection with incomplete margins (R1; 10.0% vs 2.9%; and 11.5% for APE), but the tumor was also more often staged (y)pT4 (9.2% vs 3.0%). Interestingly, in the unplanned low-HP group the DRM was more often positive in case of an R1 resection as compared to the rLAR group (70% vs 29%). Following correction for confounders in multivariable analysis, the type of procedure was not statistically associated with 4-year LR, DM, or DFS.

This is the first study that investigated the difference between planned and unplanned low-HP. While both groups had roughly similar results, this observation may oversimplify the underlying factors. The planned group typically includes patients with more advanced disease or comorbidities as compared to rLAR, which may explain their worse outcomes. The unplanned group, expected to mirror the rLAR selection, seemed to include patients encountering significant intraoperative difficulties, leading to issues such as higher DRM and CRM involvement. These factors, rather than the procedure type alone, likely drive the differences in long-term outcomes. Discrepancies with previous studies may be attributed to variations in included variables and patient selection for multivariable analysis [15, 16]. A mediation and sensitivity analysis, including patients undergoing APE and not accounting for surgical approach (minimally invasive, conversion to open, or primarily open surgery), demonstrated significantly poorer oncological outcomes for low-HP. This indicates that oncological outcomes are influenced by various procedural factors. As a result of selection bias, disentangling cause and effect remains difficult. Still, in low rectal cancer, precise planning to achieve R0 resection should be the goal and functional outcome, and the risk of an anastomosis or an end colostomy should always be secondary to that.

The reported 4-year LR rates following rLAR (6%) and low-HP (planned 14%, unplanned 13%) align with previous studies [2, 3, 16, 18]. However, in contrast to prior large retrospective cohorts, the current study found no association between low-HP and oncological outcomes in multivariable analysis [16, 18]. The paramount factor contributing to LR development is R1 resection, a finding corroborated by the present study in which involved CRM and DRM were strongly correlated with LR risk [19–21]. The R1 resection rate was highest for unplanned low-HP (12%, versus 3% in the rLAR group and 9% in the planned low-HP group), mainly as a result of a positive DRM. This can be explained by more distally located tumors in the unplanned low-HP subgroup, and in some instances, a concerted effort might have been made to avoid an APE. Nonetheless, caution should be exercised in such scenarios, emphasizing the necessity for preoperative reassessment through imaging and careful consideration of alternatives when the procedure cannot be executed as planned. Although this topic lies beyond the scope of the present study, the authors propose that an extralevator APE might be required in such cases. In our view, the exposure of the distal mesorectum might still be compromised if performing intersphincteric APE, because keeping the external sphincter with levator muscles in place might prevent adequate view on the distal TME plane in these difficult cases. Alternatively, if relevant expertise is available, a transanal TME might be considered to avoid DRM positivity given the optimized exposure to the distal TME plane.

Intraoperative technical challenges, also in the unplanned low-HP group, have likely contributed to worse oncological outcomes in univariable analysis. In unplanned low-HP cases, potential technical hurdles are reflected in prolonged surgical duration and frequent conversions from minimally invasive techniques to open surgery. Notably, this conversion is strongly associated with increased risk of LR and/or DM in multivariable analysis. While the choice between surgical techniques (low-HP or rLAR) may not inherently impact oncological outcomes, the quality of surgery, especially in the presence of technical challenges during the procedure, could significantly influence outcomes. However, directly attributing outcomes to specific procedural differences is complex. The observed worse outcomes in univariable analyses following low-HP may instead be influenced by a combination of interrelated factors. This is supported by the absence of an association between low-HP and worse outcomes in multivariable analyses, suggesting that other variables play a more prominent role in determining these results.

On the other hand, some preoperatively known factors can be used for better preoperative planning. In planned low-HP, patients had a higher cT stage but a similar percentage receiving neoadjuvant (C)RT, likely as a result of older age and more comorbidities, which may have contributed to poorer oncological outcomes. In unplanned low-HP, cT stage was comparable to rLAR cases, but tumors were more distal and patients had a higher BMI. Additionally, these patients often have a history of previous bowel resection, making them more prone to technical challenges during surgery, which may have contributed to poorer oncological outcomes. Unfortunately, scrutinizing LR cases revealed considerable heterogeneity in terms of tumor location, cT stage, surgical approach, and the site of recurrence. This illustrates the difficulties in defining the reasons for failure after unplanned low-HP.

During TME surgery, a substantial proportion of patients (35–50%) experience inadvertent retention of mesorectal tissue, indicating suboptimal treatment [22, 23]. This incomplete resection has been identified as a predictive factor for LR [24, 25]. A study on partial mesorectal excision surgery revealed that 86% of patients who developed LR exhibited residual mesorectal tissue on postoperative MRI, highlighting its role in recurrence [26]. Although MRI information regarding postoperative mesorectum was unavailable in our study, inadvertent residual mesorectum may play a role, especially in the unplanned low-HP group given the high proportion of positive DRM. Transanal TME or extralevator APE might reduce the risk of residual mesorectal tissue [23].

Several limitations warrant acknowledgement. The retrospective nature of the study inherently introduces selection bias, which has been mitigated to the best of our ability through multivariable analysis. Moreover, the resections were performed in 2016, which limits the generalizability of the findings to current practice, as surgical techniques and perioperative management may have evolved since then. Additionally, certain pertinent information, such as the rationale behind the intraoperative decision to perform low-HP, the level of experience of the operating surgeon(s), or referral to an expert center, was not captured in the dataset. The number of unplanned low-HPs among the total group was low, limiting statistical power. However, this study adds to the existing literature by describing and analyzing unplanned low-HP and planned low-HP as two distinct clinical entities.

## Conclusion

This study revealed that planned and unplanned low-HP procedures showed poorer oncological outcomes compared to rLAR in univariable analysis, but this was not associated with the type of procedure in multivariable analysis. Technical challenges encountered during unplanned low-HP have likely contributed to these poorer oncological outcomes, but it is difficult to disentangle cause and effect. Careful preoperative planning and intraoperative consideration, particularly for distal tumors, is warranted to lower DRM and CRM positivity. Although beyond the scope of this study, conversion to extralevator APE or a transanal TME rather than pursuing low-HP in case of technical difficulties may improve resection margins in selected cases. These findings underscore the importance of surgical decision-making and patient selection.

## Supplementary Information

Below is the link to the electronic supplementary material.

Supplementary file1 (DOCX 138 KB)

## Data Availability

Data will be made available upon reasonable request.
