# Supplementary material for: Oncological outcomes of planned and unplanned low Hartmann’s procedure and restorative low anterior resection for rectal cancer: a population-based cross-sectional study
Source: Tech Coloproctol. 2025 Nov 23;30(1):4. doi: 10.1007/s10151-025-03169-5 (PMC12678588; doi:10.1007/s10151-025-03169-5)
Supplement: Supplementary file 1 — Supplementary file1 (DOCX 138 KB) [file 10151_2025_3169_MOESM1_ESM.docx]

Supplementary tables & figures:

- Supplementary figure 1: flowchart of patient selection
- Supplementary table 1A-B: multivariable cox regression analyses including APE patients
- Supplementary table 2: recurrent local disease of patients with an unplanned low-HP

**Supplementary figure 1: flowchart of patient selection**

**
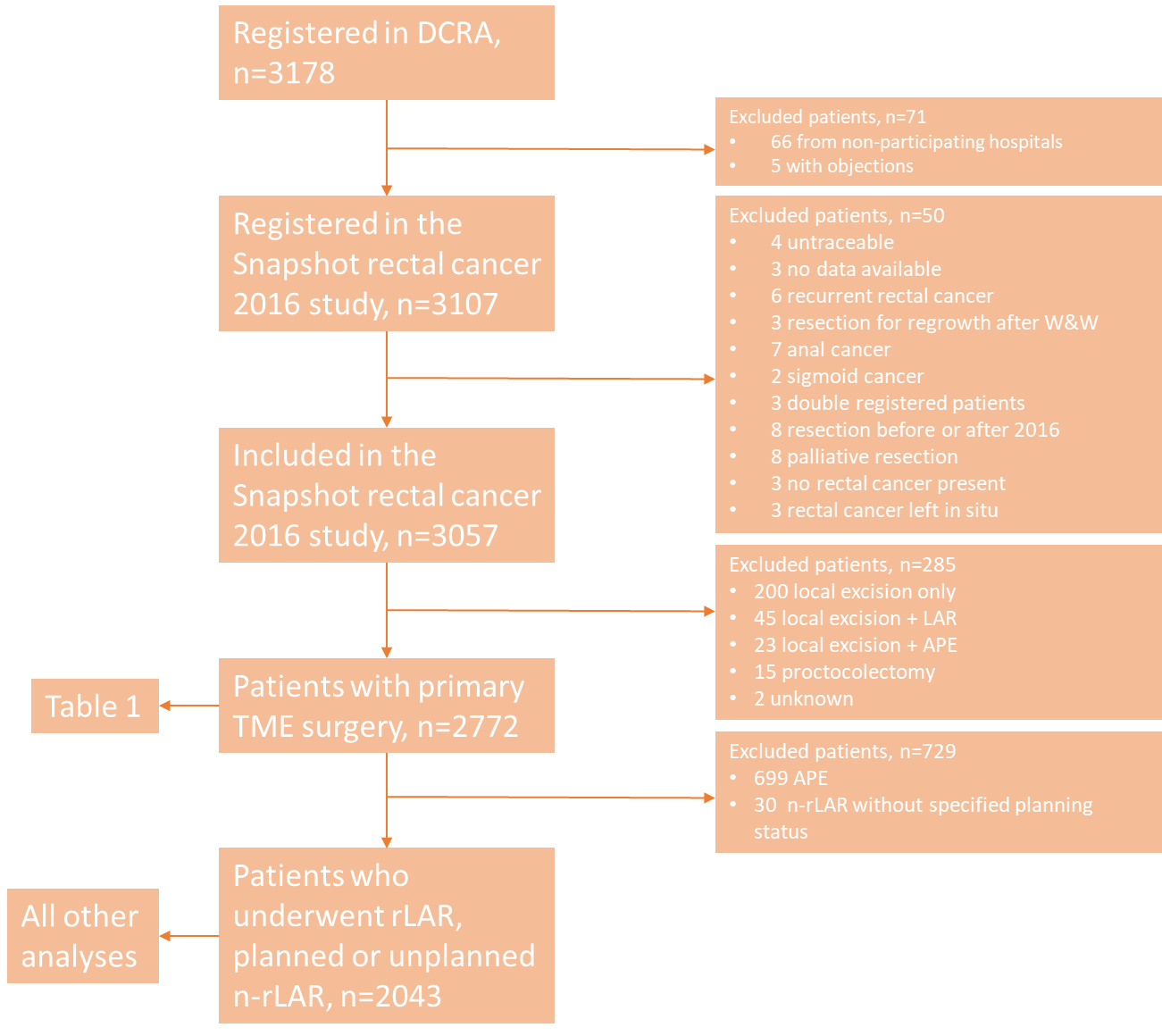
**

*Abbreviations: Dutch ColoRectal Audit, DCRA; Total Mesorectal Excision, TME; Watch and Wait, W&W; Low Anterior Resection, LAR; Abdomino Perineal Excision, AP*

| A. |  |  | Local Recurrence | | | | Distant metastases | |  | |
| --- | --- | --- | --- | --- | --- | --- | --- | --- | --- | --- |
|  |  |  | Univariable | | Multivariable | | Univariable | | Multivariable | |
|  | Variable | No. | HR (95% CI) | P | HR | P | HR (95% CI) | P | HR (95%CI) | P |
| Age | ≤65  >65 | 1188  1584 | 1.000  0.879 (0.666-1.160) | 0.361 |  |  | 1.000  0.982 (0.828-1.164) | 0.830 |  |  |
| Sex | Male  Female | 1789  983 | 1.000  0.813 (0.602-1.097) | 0.176 |  |  | 1.000  0.960 (0.804-1.147) | 0.654 |  |  |
| ASA | I/II  III/IV/V | 2255  492 | 1.000  0.881 (0.594-1.306) | 0.529 |  |  | 1.000  1.276 (1.029-1.583) | **0.026** |  | NS |
| Distance to the ARJ | ≤3 cm  3.1-7 cm  >7 cm | 785  1027  774 | 1.000  0.607 (0.443-0.832)  0.394 (0.265-0.583) | **<0.001** |  | NS | 1.000  0.612 (0.499-0.751)  0.656 (0.528-0.815) | **<0.001** |  | NS |
| cT | T1-2  T3  T4 | 764  1617  269 | 1.000  1.361 (0.956-1.936)  2.675 (1.695-4.221) | **<0.001** |  | NS | 1.000  1.700 (1.363-2.121)  3.044 (2.268-4.086) | **<0.001** |  | NS |
| MRF | Not involved  Involved | 855  1693 | 1.000  1.838 (1.373-2.461) | **<0.001** |  | NS | 1.000  1.918 (1.604-2.292) | **<0.001** | 1.000  1.453 (1.158-1.824) | **0.001** |
| cN | N0  N1  N2 | 1110  862  691 | 1.000  0.722 (0.495-1.053)  1.664 (1.208-2.291) | **<0.001** |  | NS | 1.000  1.398 (1.136-1.721)  1.670 (1.348-2.068) | **<0.001** |  | NS |
| Synchronous metastases | No  Yes | 2567  204 | 1.000  1.878 (1.170-3.016) | **0.009** |  | NS | NA |  |  |  |
| Neoadjuvant radiotherapy | None  5x5 short interval  5x5 long interval  CRT | 1071  471  298  932 | 1.000  0.312 (0.161-0.606)  2.282 (1.526-3.412)  1.408 (1.025-1.934) | **<0.001** | 1.000  0.288 (0.148-0.560)  1.540 (1.019-2.340)  1.122 (0.805-1.564) | **<0.001** | 1.000  1.324 (1.018-1.722)  3.229 (2.474-4.213)  1.794 (1.456-2.211) | **<0.001** | 1.000  1.284 (0.970-1.698)  2.388 (1.763-3.237)  1.267 (0.973-1.649) | **<0.001** |
| Type of resection | rLAR  APE  low-HP | 1704  683  385 | 1.000  2.102 (1.534-2.881)  2.500 (1.732-3.606) | **<0.001** | 1.000  1.390 (0.987-1.957)  1.922 (1.325-2.789) | **0.002** | 1.000  1.917 (1.589-2.313)  1.539 (1.197-1.979) | **<0.001** | 1.000  1.378 (1.115-1.702)  1.180 (0.897-1.554) | **0.012** |
| Multivisceral resection | No  Yes | 2557  215 | 1.000  3.181 (2.209-4.583) | **<0.001** | 1.000  1.703 (1.145-2.532) | **0.009** | 1.000  2.212 (1.697-2.883) | **<0.001** |  | NS |
| Duration of surgery | ≤ 200 min  > 200 | 1159  1002 | 1.000  1.691 (1.237-2.311) | **<0.001** |  | NS | 1.000  1.328 (1.099-1.604) | **0.003** |  | NS |
| Pelvic sepsis | No  Yes | 2343  429 | 1.000  1.725 (1.244-2.392) | **0.001** | 1.000  1.654 (1.189-2.301) | **<0.001** | 1.000  1.225 (0.980-1.530) | 0.075 |  | NS |
| Blood transfusion | No  Yes | 2414  221 | 1.000  1.716 (1.091-2.701) | **0.020** |  | NS | 1.000  1.856 (1.403-2.456) | **<0.001** | 1.000  1.454 (1.079-1.959) | **0.014** |
| Margin status | R0  R1/R2 | 2604  166 | 1.000  6.854 (4.925-9.539) | **<0.001** | 1.000  5.009 (3.497-7.174) | **<0.001** | 1.000  3.725 (2.886-4.809) | **<0.001** | 1.000  2.690 (2.032-3.560) | **<0.001** |

**Supplementary table 1A-B: Multivariable cox regression analyses including APE patients**

**Abbreviations**: ASA, American Society of Anesthesiologists; ARJ, anorectal junction; cT-stage, clinical T-stage, MRF, mesorectal fascia; cN-stage, clinical N-stage; rLAR, restorative lower anterior resection; APE, abdominoperineal excision, n-rLAR, non-restorative lower anterior resection; MI, minimally invasive; HR, hazard ratio.

| B. |  |  | Disease free survival | | | | Overall survival | |  | |
| --- | --- | --- | --- | --- | --- | --- | --- | --- | --- | --- |
|  |  |  | Univariable | | Multivariable | | Univariable | | Multivariable | |
|  | Variable | No. | HR | P | HR | P | HR (95% CI) | P | HR (95%CI) | P |
| Age | ≤65  >65 | 1171  1569 | 1.000  1.167 (1.026-1.329) | **0.019** |  | NS | 1.000  1.691 (1.425-2.008) | **<0.001** | 1.000  1.629 (1.349-1.968) | **<0.001** |
| Sex | Male  Female | 1762  978 | 1.000  0.925 (0.809-1.057) | 0.252 |  |  | 1.000  0.769 (0.769-1.082) | 0.290 |  |  |
| ASA | I/II  III/IV/V | 2224  491 | 1.000  1.527 (1.314-1.774) | **<0.001** | 1.000  1.321 (1.115-1.564) | **0.001** | 1.000  2.018 (1.685-2.416) | **<0.001** | 1.000  1.437 (1.175-1.757) | **<0.001** |
| Distance to the ARJ | ≤3 cm  3.1-7 cm  >7 cm | 769  1016  769 | 1.000  0.702 (0.602-0.819)  0.703 (0.596-0.829) | **<0.001** |  | NS | 1.000  0.641 (0.527-0.781)  0.688 (0.559-0.847) | **<0.001** |  | NS |
| cT | T1-2  T3  T4 | 755  1602  261 | 1.000  1.788 (1.510-2.118)  3.093 (2.474-3.866) | **<0.001** | 1.000  1.458 (1.190-1.788)  1.697 (1.277-2.254) | **<0.001** | 1.000  1.877 (1.501-2.348)  3.221 (2.424-4.279) | **<0.001** | 1.000  1.577 (1.222-2.035)  1.920 (1.374-2.681) | **<0.001** |
| MRF | Not involved  Involved | 839  1678 | 1.000  1.898 (1.660-2.169) | **<0.001** | 1.000  1.284 (1.076-1.532) | **0.006** | 1.000  1.842 (1.544-2.184) | **<0.001** |  | NS |
| cN | N0  N1  N2 | 1093  858  681 | 1.000  1.424 (1.215-1.667)  1.802 (1.534-2.115) | **<0.001** |  | NS | 1.000  1.290 (1.056-1.575)  1.481 (1.207-1.818) | **<0.001** |  | NS |
| Synchronous metastases | No  Yes | 2536  204 | NA |  |  |  | 1.000  4.855 (3.996-5.973) | **<0.001** | 1.000  3.207 (2.550-4.032) | **<0.001** |
| Neoadjuvant radiotherapy | None  5x5 short interval  5x5 long interval  CRT | 1057  470  292  921 | 1.000  1.041 (0.846-1.281)  3.387 (2.809-4.084)  1.640 (1.404-1.917) | **<0.001** | 1.000  0.991 (0.789-1.246)  2.212 (1.753-2.791)  1.093 (0.883-1.353) | **<0.001** | 1.000  0.880 (0.668-1.160)  3.254 (2.592-4.085)  1.444 (1.184-1.761) | **<0.001** | 1.000  0.861 (0.640-1.157)  1.607 (1.217-2.121)  1.047 (0.819-1.337) | **<0.001** |
| Type of resection | rLAR  APR  low-HP | 1687  675  378 | 1.000  1.813 (1.571-2.092)  1.865 (1.565-2.221) | **<0.001** | 1.000  1.314 (1.115-1.549)  1.227 (1.005-1.498) | **0.003** | 1.000  2.028 (1.688-2.436)  2.497 (2.020-3.087) | **<0.001** | 1.000  1.473 (1.197-1.812)  1.546 (1.220-1.960) | **<0.001** |
| Multivisceral resection | No  Yes | 2532  208 | 1.000  2.303 (1.902-2.790) | **<0.001** |  | NS | 1.000  2.520 (2.007-3.164) | **<0.001** |  | NS |
| Duration of surgery | ≤ 200 min  > 200 | 1149  990 | 1.000  1.347 (1.168-1.553) | **<0.001** |  | NS | 1.000  1.236 (1.030-1.484) | **0.023** |  | NS |
| Pelvic sepsis | No  Yes | 2318  422 | 1.000  1.363 (1.160-1.601) | **<0.001** | 1.000  1.278 (1.071-1.526) | 0.007 | 1.000  1.363 (1.113-1.670) | **0.003** | 1.000  1.341 (1.076-1.670) | NS |
| Blood transfusion | No  Yes | 2386  218 | 1.000  2.247 (1.859-2.716) | **<0.001** | 1.000  1.534 (1.240-1.899) | **<0.001** | 1.000  3.011 (2.421-3.745) | **<0.001** | 1.000  1.688 (1.324-2.151) | **<0.001** |
| Margin status | R0  R1/R2 | 2578  160 | 1.000  3.5258 (2.910-4.277) | **<0.001** | 1.000  2.521 (2.029-3.133) | **<0.001** | 1.000  4.038 (3.231-5.048) | **<0.001** | 1.000  2.510 (1.963-3.208) | **<0.001** |

**Abbreviations**: ASA, American Society of Anesthesiologists; ARJ, anorectal junction; cT-stage, clinical T-stage, MRF, mesorectal fascia; cN-stage, clinical N-stage; rLAR, restorative lower anterior resection; APE, abdominoperineal excision, n-rLAR, non-restorative lower anterior resection; MI, minimally invasive; HR, hazard ratio.

| No. | Distance to the ARJ (≤ 3cm) | cT-stage (incl MRF) | mrEMVI | Neoadjuvant therapy | Initial approach | Conversion | Pelvic sepsis | pT-stage | R1 resection | LR location | Metachronous metastases |
| --- | --- | --- | --- | --- | --- | --- | --- | --- | --- | --- | --- |
| 1 | + | 3 (MRF-) | Grade 3 | CRT | Open | NA | - | 3 | + (DRM) | Presacral | - |
| 2 | + | 3 (MRF-) | Not present | CRT | MI | - | + | 3 | - | Rectal stump | - |
| 3 | + | 3 (MRF-) | Not present | - | MI | + | + | 2 | - | Rectal stump | + (metachr. LR) |
| 4 | - | 3 (MRF-) | Missing | - | MI | + | - | 3 | - | Rectal stump | + (synchr. LR) |
| 5 | - | 3 (MRF-) | Missing | CRT | Open | NA | - | 3 | - | Lateral | + (synchr. LR) |
| 6 | + | 4 (MRF+) | Grade 4 | RT | MI | - | - | 3 | - | Unknown | + (synchr. LR) |
| 7 | - | 4 (MRF+) | Grade 4 | - | MI | + | - | 3 | - | Multifocal | + (synchr. LR) |
| 8 | - | 3 (MRF+) | Grade 3 | CRT | MI | - | - | 3 | - | Presacral | + (synchr. LR) |
| 9 | - | 3 (MRF+) | Not present | RT | TaTME | - | + | 3 | - | Lateral | + (synchr. LR) |

Supplementary table 2: Recurrent local disease of patients with an unplanned low-HP

**Abbreviations**: ARJ, anorectal junction; cT-stage, clinical T-stage; MRF, mesorectal fascia; mrEMVI, MRI-detected extramural venous invasion; CRT, chemoradiation therapy; RT, radiotherapy; MI, minimally invasive; TaTME, transanal total mesorectal excision; NA not applicable; pT-stage, pathological T-stage; DRM, distal resection margin; LR, local recurrence.
